# Lumen-Apposing Metal Stent in the Treatment of Persistent Dysphagia due to Cervical Radiation Therapy

**DOI:** 10.5152/tjg.2026.26290

**Published:** 2026-09-03

**Authors:** Eren Yilmaz, Mucahit Ergul, Oguz Ozturk, Kadir Can Acun, Emir Tuğrul Keskin, Orhan Coskun, Ilhami Yuksel

**Affiliations:** 1 Department of Gastroenterology, Ankara Bilkent City Hospital, Ankara, Türkiye; 2 Department of Gastroenterology, Ankara Yıldırım Beyazıt University School of Medicine, Ankara, Türkiye

## Case Presentation

Esophageal strictures are generally classified as either malignant and benign. The most common causes of benign strictures include gastroesophageal reflux disease, radiation therapy, caustic ingestion, surgical anastomoses, and endoscopic interventions. Compared with malignant strictures, benign strictures are more amenable to endoscopic treatment.^1^ The most commonly used treatment modalities for benign esophageal strictures are bougie or balloon dilation.^2^ Fully covered self-expanding metal stents (FCSEMSs) and lumen-apposing metal stents (LAMSs) can be utilized in the management of benign esophageal strictures.

A 61-year-old man with a history of surgery and cervical radiation therapy presented with dysphagia. Written consent was obtained. A barium swallow demonstrated a short 2-cm cervical esophageal stricture (Figure 1). On endoscopy, a stricture was identified 12 cm from the incisors (Figure 2). Monthly endoscopic dilatation was required because of persistent symptoms. Because a short-segment stricture required frequent endoscopic dilations, temporary stent placement was considered. LAMS was selected because its wide anchoring flanges were expected to minimize the risk of migration. LAMS (14 × 20 mm; Micro-Tech, Nanjing, China) was placed across the stricture using a dual-channel gastroscope (Fujinon EG-530D; FujifilmCorporation, Tokyo, Japan) under endoscopic guidance (Figure 3). After LAMS placement, luminal patency was confirmed fluoroscopically using a water-soluble contrast medium, and the esophageal stricture was observed to be open. Six hours later, the patient was able to tolerate a soft diet. Five weeks later, LAMS was removed, and endoscopy showed prominent dilation of the stenotic segment (Figure 4). The patient was able to tolerate liquids and a soft diet.

## Technique

Endoscopic dilation remains the standard treatment for esophageal strictures, providing rapid and effective improvement in dysphagia. In patients with refractory esophageal strictures who fail endoscopic dilation, adjunctive treatments, including intralesional steroid or mitomycin C injection and endoscopic stent placement, may be considered.^3^ The optimal treatment strategy should be individualized according to the etiology and complexity of the stricture, as well as the patient’s response to previous treatment modalities. In patients with refractory esophageal strictures, esophageal stent placement may be considered as a therapeutic option. Stent placement can maintain luminal patency and reduce the frequency of subsequent dilation sessions. Both FCSEMSs and LAMSs are available for esophageal stenting. The reported clinical success rate of these stents is approximately 40%, whereas stent migration has been reported in nearly 30% of cases.^3^ Despite modest success rates, esophageal stenting remains a valuable therapeutic option for patients requiring frequent or repeated endoscopic dilation. The risk of serious long-term complications associated with these stents has prompted interest in biodegradable stents. These stents undergo spontaneous degradation through hydrolysis, eliminating the need for endoscopic removal and potentially reducing long-term metallic stent complications. Although these stents are theoretically expected to provide efficacy comparable to that of FCSEMSs, reported clinical success rates have varied across studies. Their widespread use remains limited by their high cost and restricted availability.^4^ Although the upfront cost of LAMS placement is substantially higher than that of a single endoscopic dilation, it may represent an economically reasonable alternative in carefully selected patients requiring frequent repeated dilation sessions, potentially reducing the number of subsequent endoscopic interventions.^5^

LAMSs have a short length and wide anchoring flanges that facilitate lumen-to-lumen apposition, thereby reducing the risks of migration, leakage, and related adverse events. Their larger diameter, compared with those of plastic and conventional metal biliary stents, enables direct endoscopic interventions. In addition, the fully silicone-covered nitinol framework facilitates stent removal and prevents tissue ingrowth.^6^^,^^7^ Because of their unique design, LAMSs have a lower risk of stent migration. Their relatively short length makes them particularly suitable for treating short-segment strictures.

Although the bilateral flanged design of LAMS lowers the risk of stent migration, careful placement is important, particularly in cervical strictures, where optimal positioning may be challenging. Placement of LAMS in close proximity to the upper esophageal sphincter may cause odynophagia, patient discomfort, pressure-induced mucosal ulceration and bleeding. Prolonged stent dwell time may result in flange embedment and tissue ingrowth. In rare cases, it may also lead to perforation and fistula formation.^8^ Therefore, LAMS should not be left in place for longer than 12 weeks.^7^^,^^8^

The LAMS was removed at week 5. During this period, the patient tolerated a liquid diet without difficulty but required analgesic therapy for pain management. Upon stent removal, mild ulceration and granulation tissue formation were observed; however, there was no evidence of bleeding or fistula formation. Importantly, stent migration, which is frequently encountered with other fully covered stents, was not observed.

## Conclusion

LAMS may offer safe therapeutic option to bypass short-diameter refractory cervical esophageal strictures, offering an alternative to feeding tubes, endoscopic dilation, standard metal stents, or surgery in patients with upper esophageal refractory dysphagia (Video 1).

## Figures and Tables

**Figure 1.**
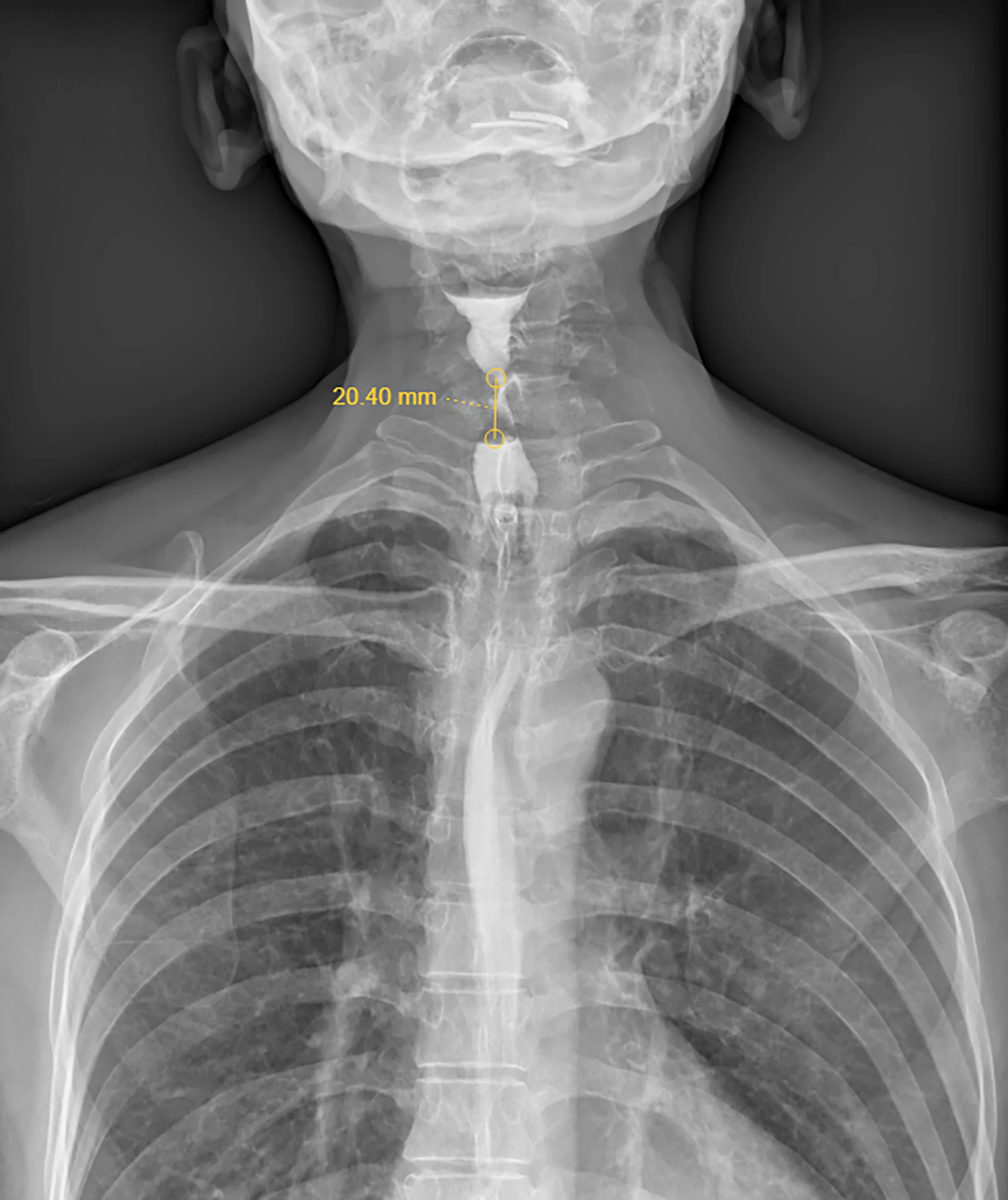
A barium swallow study demonstrated an esophageal stricture.

**Figure 2.**
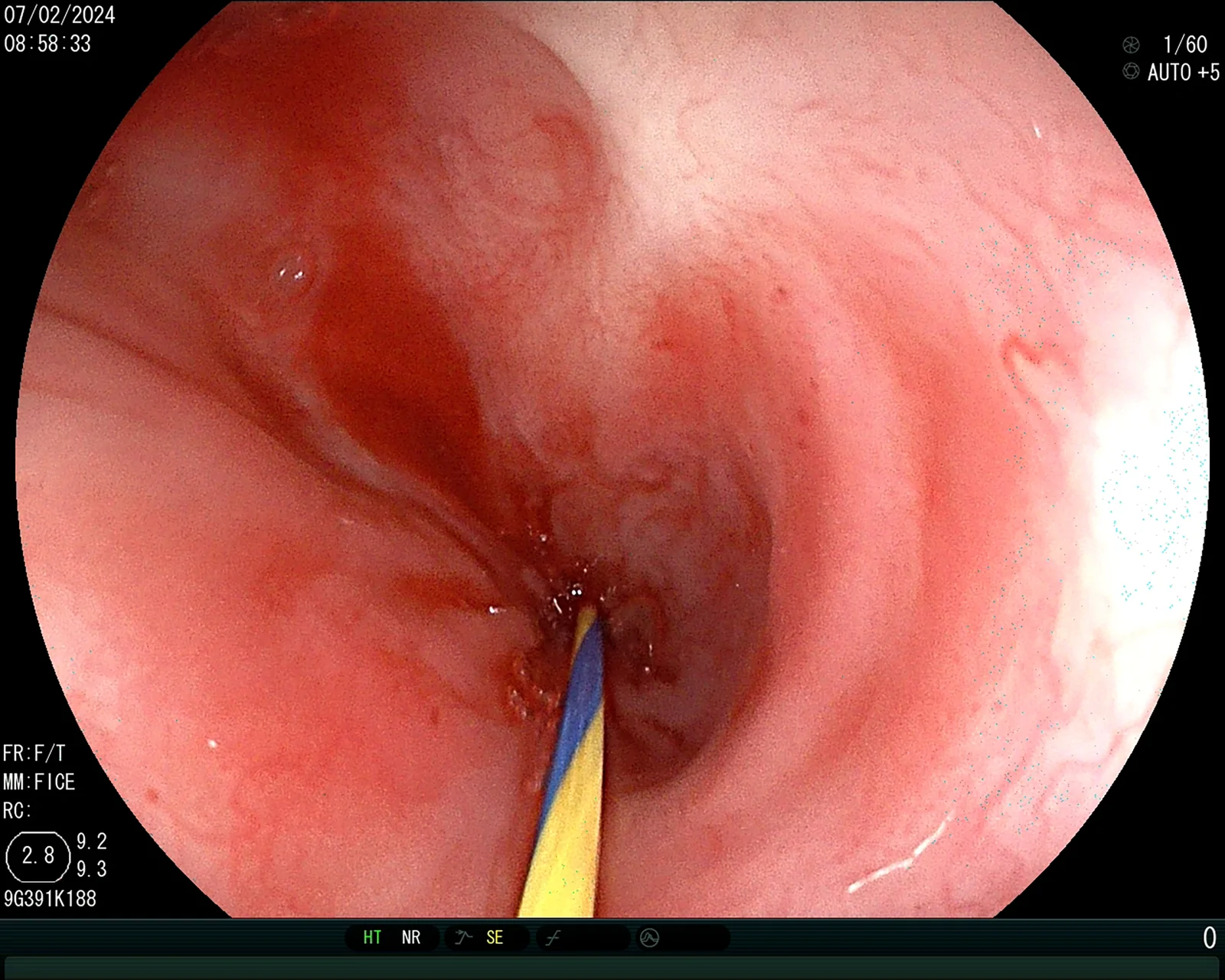
A guidewire was advanced across the esophageal stricture under fluoroscopic guidance.

**Figure 3.**
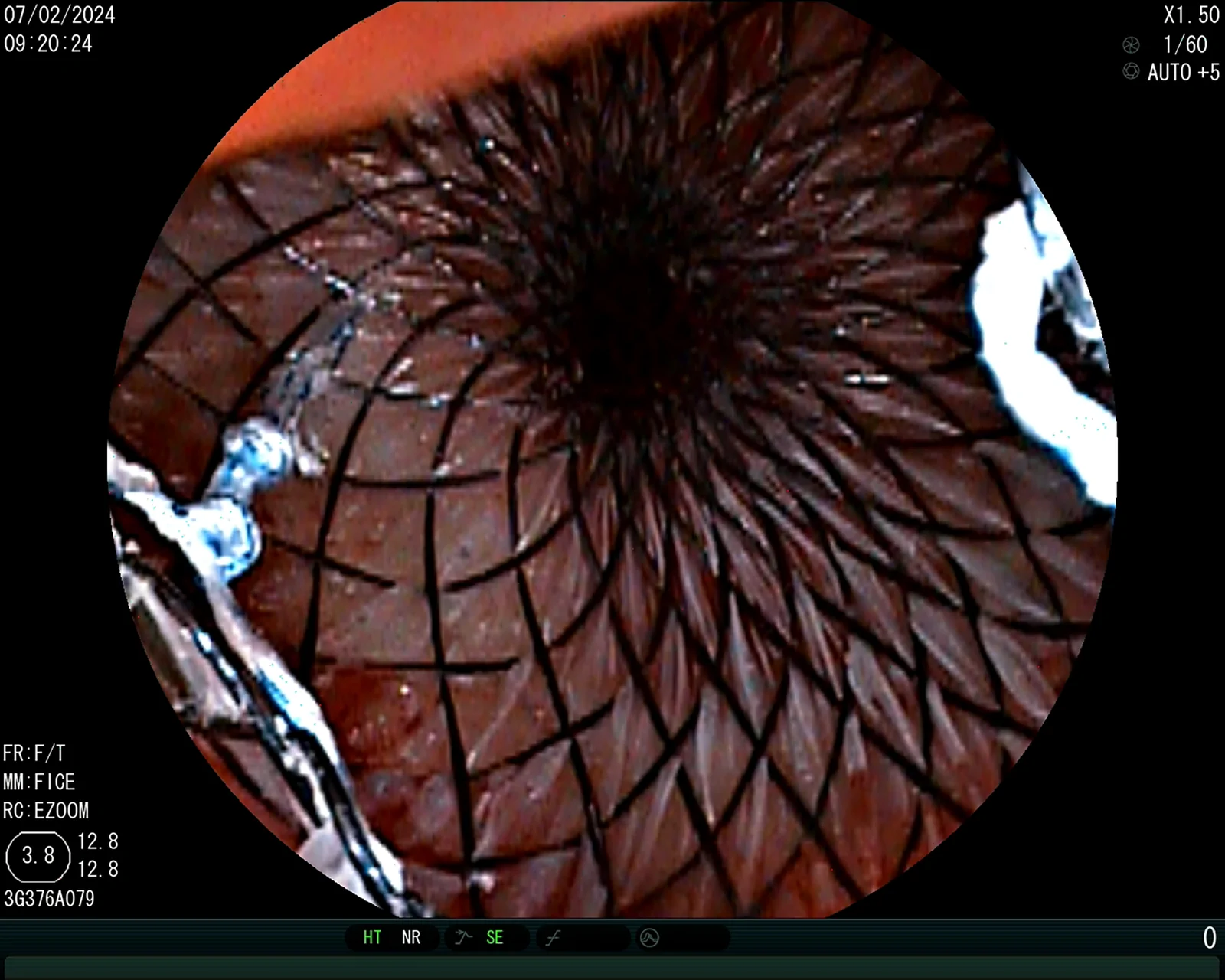
Luminal patency was achieved in the esophagus following LAMS placement. LAMS, lumen-apposing metal stent.

**Figure 4.**
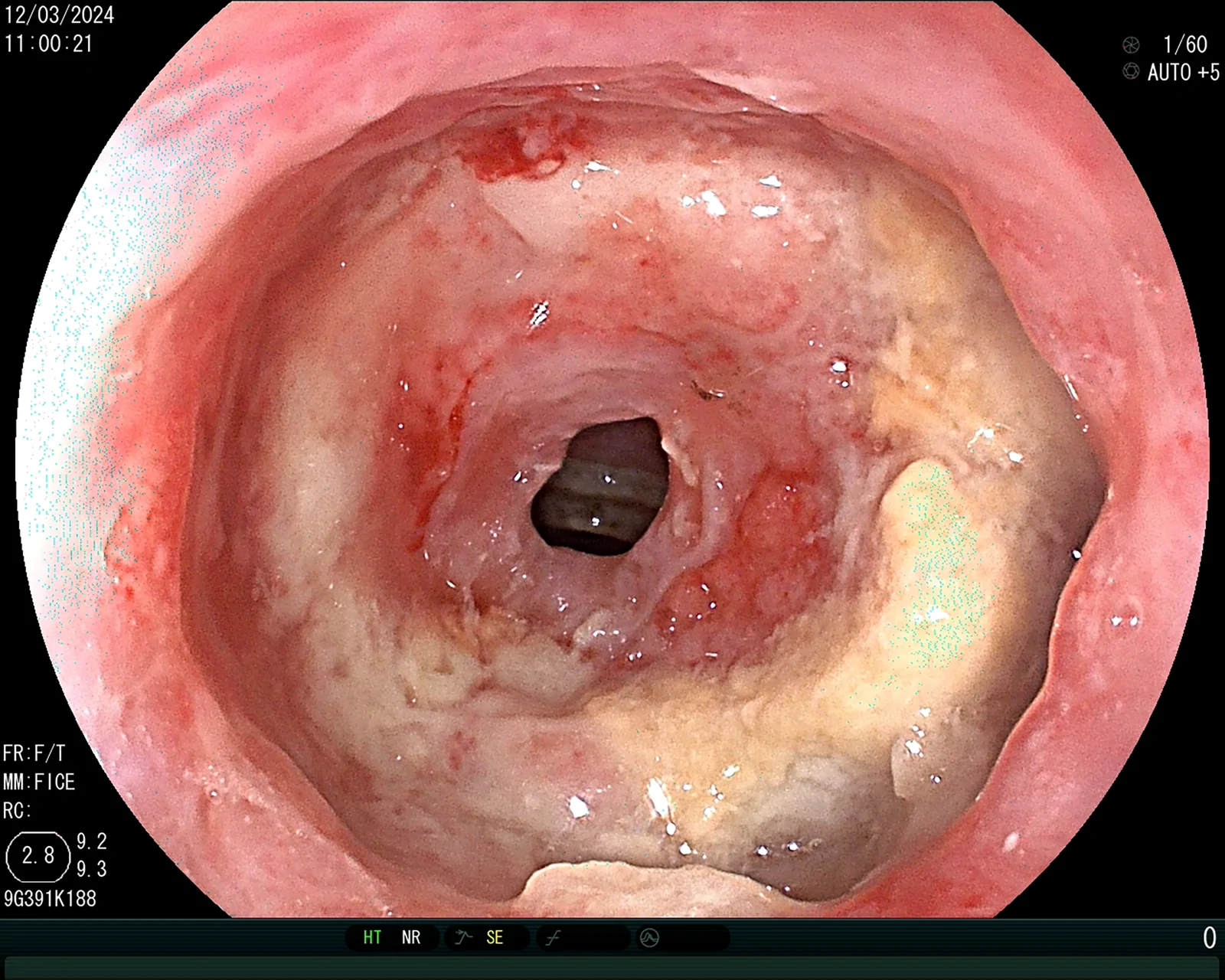
Following LAMS removal, luminal patency was maintained, with surrounding granulation tissue observed. LAMS, lumen-apposing metal stent.

## Data Availability

The data that support the findings of this study are available on request from the corresponding author.
